# The First Three Mitochondrial Genomes for the Characterization of the Genus *Egeirotrioza* (Hemiptera: Triozidae) and Phylogenetic Implications

**DOI:** 10.3390/genes15070842

**Published:** 2024-06-26

**Authors:** Zhulidezi Aishan, Ze-Lu Mu, Zi-Cong Li, Xin-Yu Luo, Ning Huangfu

**Affiliations:** 1 Xinjiang Key Laboratory of Biological Resources and Genetic Engineering, College of Life Science and Technology, Xinjiang University, Urumqi 830017, China; zhulidezi.a@xju.edu.cn (Z.A.); 107552301054@stu.xju.edu.cn (Z.-L.M.); 107552100946@stu.xju.edu.cn (Z.-C.L.); 2 Department of Entomology, China Agricultural University, Beijing 100193, China; ltquail@126.com; 3 National Natural History Museum of China, Beijing 100050, China

**Keywords:** Psylloidea, mitochondrial genome, *Populus euphratica*, phylogenomics

## Abstract

(1) Background: Mitochondrial genomes are important markers for the study of phylogenetics and systematics. Triozidae includes some primary pests of *Populus euphratica*. The phylogenetic relationships of this group remain controversial due to the lack of molecular data. (2) Methods: Mitochondria of *Egeirotrioza* Boselli were sequenced and assembled. We analyzed the sequence length, nucleotide composition, and evolutionary rate of Triozidae, combined with the 13 published mitochondrial genomes. (3) Results: The evolutionary rate of protein-coding genes was as follows: *ATP8* > *ND6* > *ND5* > *ND2* > *ND4* > *ND4L* > *ND1* > *ND3* > *APT6* > *CYTB* > *COX3* > *COX2* > *COX1*. We reconstructed the phylogenetic relationships of Triozidae based on 16 triozid mitochondrial genomes (thirteen ingroups and three outgroups) using the maximum likelihood (ML) and Bayesian inference (BI) approaches. The phylogenetic analysis of the 16 Triozidae mitochondrial genomes showed that *Egeirotrioza* was closely related to *Leptynoptera*. (4) Conclusions: We have identified 13 PCGs, 22 tRNAs, 2 rRNAs, and 1 control region (CR) of all newly sequenced mitochondrial genomes, which were the mitochondrial gene type in animals. The results of this study provide valuable genomic information for the study of psyllid species.

## 1. Introduction

Mitochondrial genomes, as one of the important molecular markers, are usually used for studies on speciation, phylogeography, evolutionary history, and phylogeny in insect groups [1,2,3], benefiting from easy availability, maternal inheritance, and high substitution rates [3,4]. Meanwhile, insect mitochondrial genomes’ structural characteristics are conserved, and they can also offer additional evidence of morphological classification. The insect mitochondrial genome ranges mostly from 14,000 to 20,000 bp in length [3], containing 2 ribosomal RNAs (rRNAs), 13 protein-coding genes (PCGs), 22 transfer RNA (tRNAs), and 1 non-coding control region (CR) [4]. Mitochondrial genomes are often referred to as the secondary genetic information system due to their distinctive genetic characteristics [5].

Psyllids (Psylloidea), or jumping plant lice, are an important group within the suborder Sternorrhyncha (Hemiptera), containing more than 4000 described species [6]. All species of Sternorrhyncha are primarily pests, such as whiteflies, aphids, and scales, and they directly inflict damage on host plants through feeding or gall development [7]. During the immature stages, species of psyllids exhibit highly host-specific behavior [8]. These pests not only cause direct damage by siphoning plant sap but also act as vectors for the transmission of plant viruses, inflicting substantial economic losses on agriculture and forestry crops.

Triozidae is a family within Psylloidea. This family comprises about 1000 described species and 70 genera [9]. There are many synonymous genera and species of psyllids, and most phylogenetic studies of Psyllioidea have been based on morphological research, but there has been no evidence of molecular correlation. As Egeirotrioza has never been reported as a molecular marker, the genus was not included in previous phylogenetic studies based on molecular morphology. Some species of this group are the main pests of *P. euphratica*. Some psyllid species, in order to create a good, stable growth and development environment, can make galls, but psyllid galls are mainly deposited on the leaves of the host plant. A gall is the abnormal tissue formed by the plant when stimulated through psyllid feeding or egg-laying; the nymphs live in the galls as adults [10]. *P. euphratica* is an important forest resource in desert areas, playing a crucial role in windbreak and sand fixation, regulating the climate of oases, and stabilizing the ecological balance in desert zones [10,11]. Investigations into the endangered situation of *P. euphratica* have revealed significant damage caused by psyllids in northern and southern Xinjiang [12].

**Figure 1:**
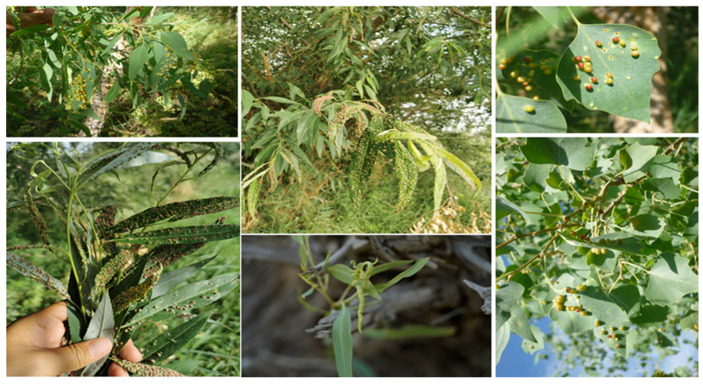
*Populus euphratica* leaves covered with galls.

We conducted a field survey of poplar forests in Luntai County and Shaya County, Xinjiang, China, from 2021 to 2023. Through field research, we found that infestation rates on new shoots of saplings often exceed 80% (unpublished), with a high propensity for rapid dissemination, leading to widespread infestations that result in seedling mortality and wilting and thereby causing catastrophic impacts on the growth and development of *P. euphratica*. In Xinjiang, both natural and artificially planted *P. euphratica* forests are affected to varying extents by psyllids, including *Egeirotrioza rufa* and *Egeirotrioza gracilis*, leading to poor growth and abnormal development in young trees. During psyllid outbreaks, when pest density is high, *P. euphratica* leaves are covered with galls on both sides, and severe infestations lead to premature leaf yellowing, resulting in weakened tree vigor and slow growth (Figure 1). These pests not only harm *P. euphratica* but also threaten surrounding species such as tamarisk and Haloxylon, posing a threat to the desert forests of Xinjiang and the ecological environment of western China’s desert regions.

Despite Triozidae being important pests, molecular data, and especially mitochondrial genome resources, on Triozidae are still deficient. To date, only a few mitochondrial genomes in this group have been made available on GenBank and other databases. The lack of molecular data seriously restricts our understanding of biological characteristics’ evolution.

In this study, we newly sequenced, assembled, and annotated three species of *Egeirotrioza* within Triozidae and also analyzed the features of their mitochondrial genome structure. Combined with 10 previously published mitochondrial genomes among Triozidae, we compared the substitution, main features, and evolutionary rates. Finally, we reconstructed the phylogenetic relationships of Triozidae based on 16 mitochondrial genomes (13 ingroups and 3 outgroups) via maximum likelihood (ML) and Bayesian inference (BI) approaches.

## 2. Materials and Methods

### 2.1. Taxon Sampling and Sequencing 

In this study, we analyzed 16 taxa of Triozidae. We newly sequenced three species within *Egeirotrioza* which were collected from Luntai County, Xinjing, China (84.3081° E, 41.5911° N), by sweeping (detailed information shown in Table 1). In addition, the mitochondrial genomes of 13 Triozidae species were downloaded from GenBank for phylogenetic and comparative mitogenomic analyses. We selected three species that are closely related to Triozidae as outgroups. All species were identified by Xin-yu Luo, and the voucher specimen was deposited at the College of Life Science and Technology, Xinjing University, Xinjiang, China. The samples were stored in 95% ethanol at −20 °C until morphological examination and DNA extraction.

Legs were used to extract the whole genomic DNA using a Qiagen DNeasy Blood & Tissue Kit (Qiagen, Dusseldorf, Germany) according to the manufacturer’s protocol. The Qubit^®^ DNA Assay Kit in a Qubit^®^ 2.0 Flurometer (ThermoFisher, Waltham, MA, USA) was applied to measure the concentration of the DNA (Berry Genomics, Beijing, China). The Illumina NovaSeq 6000 (Illumina, San Diego, CA, USA) platform was used to generate sequencing libraries of 150 bp paired-end reads with an insert size of 350 bp. Trimmomatic v0.32 [13] was applied to remove short reads, adapters, and low-quality reads of raw data.

### 2.2. Assembly, Annotation, and Composition Analyses

Two methods were used for de novo assembly: (1) NOVOPlasty v3.8.3 (Brussels, Belgium) [14] was utilized to assemble Illumina reads and k-mer sizes of 23–39 bp; (2) IDBA-UD v1.1.3 (Boston, MA, USA) [15] was implemented for mitochondrial genome assembly (“–mink 40 –maxk 120”). Mitochondrial genome sequences were compared, which were obtained by the aforementioned methods, using Geneious 2020.2.1 [16] and merged into a single sequence. Then, tRNAscan SE 2.0 [17] and MITOS WebServer were utilized to annotate and analyze the secondary structure of tRNAs. Clustal Omega in Geneious was applied to annotate rRNAs and PCGs based on *Bactericera cockerelli*. The boundaries of rRNAs and PCGs were manually proofread using MEGA X software [18]. SeqKit v0.16.0 (Chongqing, China) [19] was used to examine the bias of the nucleotide composition and the nucleotide composition of each gene. AT-skew and GC-skew were calculated by two formulas: AT-skew = (A − T)/(A + T); GC-skew = (G − C)/(G + C). The base composition, AT- and GC-skew, and relative synonymous codon usage (RSCU) of 10 species of Nitidulidae were calculated using MEGA X. The nucleotide diversity (Pi) of 13 PCGs and 2 rRNAs of three *Egeirotrioza* species was calculated using DnaSP v6.0 with a sliding window and a step size window of 200 bp and 20 bp, respectively.

DnaSP 6.0 [20] was utilized to calculate the non-synonymous substitution rate (Ka)/synonymous substitution rate (Ks) for each PCG. CGview (https://cgview.ca/, accessed on 28 February 2024), an online server, was used to generate the visual sequence features of the mitochondrial genomes. Finally, all newly sequenced mitochondrial genomes were submitted to GenBank (for accession number).

### 2.3. Phylogenetic Analyses

To reconstruct the phylogenetic relationship of Triozidae, we sampled 13 Triozidae taxa, including species of *Trioza*, *Aacanthocnema*, *Pariaconus*, *Leptynoptera*, *Bactericera*, and *Paratrioza*. Based on the published phylogenies of Psylloidea [6], we selected two *Diphorina* species and one *Arytainilla* species as outgroups. The phylogenetic analyses of Triozidae were conducted using 2 rRNAs and 13 PCGs from 16 mitochondrial genomes. MAFFT v7.450 (Osaka, Japan) [21] was applied to align nucleotide and protein sequences with the L-INS-I method. Trimal v1.4.1 (Barcelona, Spain) [22] was utilized for sequence trimming with the “-automated1” strategy. FASconCAT-G v1.04 (Santa Cruz, CA, USA) [23] was used to concatenate the matrices finally for phylogeny analysis: (1) the cds_faa matrix, including all PCG amino acid reads; (2) the cds_fna matrix, containing all PCG nucleotide reads; (3) the cds_rrna matrix, including all PCG and two rRNA nucleotide reads; (4) the cds12_fna matrix, containing all PCG nucleotide reads except the third codon positions; (5) the cds12_rrna matrix, including all PCG nucleotide reads with the third codon positions removed and two rRNA genes. Heterogeneity among the matrices was calculated via AliGROOVE v1.06 (Bonn, Germany) [24]. 

Here, we used the ML and BI methods to reconstruct the phylogenetic relationship within Triozidae. In the ML analysis, ModelFinder [25] in IQ-TREE 2 (Canberra, ACT, Australia) [26] was used to select befitting substitution models. To minimize long-branch attraction artifacts, we also used the posterior mean site frequency (PMSF) [27] model (‘−m − mtART + C60 + FO + R’) in IQ-TREE for the cds_faa matrix. A BI tree was generated via PhyloBayes-MPI (Montréal, QC, Canada) [28] with the site-heterogeneous mixture model (−m CAT + GTR). An available online website, iTOL, was utilized to fine-tune the final phylogenetic tree (https://itol.embl.de/upload.cgi, accessed on 15 February 2024).

## 3. Results and Discussion

### 3.1. Mitogenomic Organization

**Figure 2:**
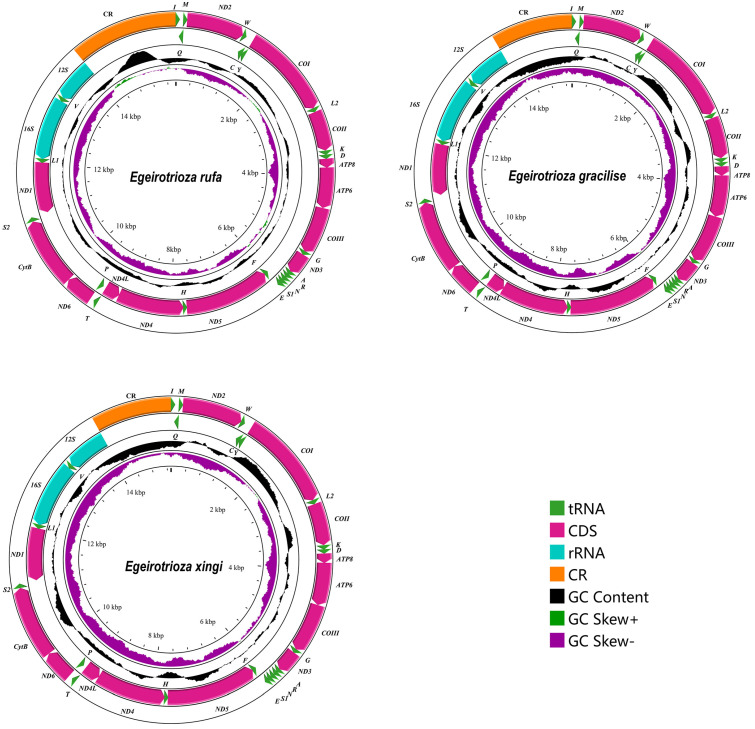
Mitochondrial genome map showing the mitochondrial genome characteristics of representative species within the genus *Egeirotrioza*. The arrows indicate the direction of gene transcription. Normative abbreviations are used to represent PCGs and rRNAs, and single letter abbreviations are used to represent tRNAs. Red, green, blue, and orange represent PCGs, tRNA, rRNA, and CR, respectively. The GC content of the complete mitochondrial genome is shown in the second circle. The GC-skew of the complete mitochondrial genome is shown in the third circle. The innermost circle shows the length of the complete mitochondrial genome.

Here, we sequenced approximately 6 GB of raw data for each species. A total of three mitochondrial genomes of Triozidae were obtained, of which two were complete mitochondrial genomes (*E. xingi* and *E. rufa*), while that of *E. gracilis* was a linear mitochondrial genome; all of them have been deposited in GenBank with the accession numbers PP471964-PP471966. The whole lengths of the newly sequenced genomes are as follows: *E. rufa*, 15,830 bp; *E. gracilis*, 15,355 bp; *E. xingi*, 15,301 bp (Table 2). The unstable size of the CR was the primary reason for the whole length, as previously observed from other triozid species [6]. We identified 13 PCGs, 22 tRNAs, 2 rRNAs, and 1 CR, which were the typeset mitochondrial genes in animals (Figure 2), and the three mitochondrial genomes exhibit a high degree of conservation. Our newly assembled mitochondrial genomes are similar to those of previously published species of Hemiptera and other insects in length and gene order [2,3,6,29,30,31]. The mitochondrial features of the represented species are depicted in Figure 2. The trnS1 secondary structures of the three mitochondrial genome sequences of *Egeirotrioza* species lack stem–loop structures with dihydrouracil, and the other 21 tRNA secondary structures are typical clover structures. The stem–loop structure with dihydrouracil deletion of trnS1 is a typical feature in insects’ mitochondrial genomes [6].

The newly sequenced mitochondrial genomes were found to have similar nucleotide compositions (Table 2), revealing the characteristic AT-biased composition in Triozidae and other insects [3,31,32]. The AT contents (%) of the three newly reported genomes are 76.92 (*E. rufa*), 74.46 (*E. gracilis*), and 76.40 (*E. xingi*) (Table 2). All the newly reported mitochondrial genomes have a positive AT-skew, while the GC-skew is negative. In most hemipteran species, there are fewer T than A and fewer G than C bases. The AT-skew and GC-skew values of the three newly reported genomes are 0.010 and −0.154 for *E. rufa*, −0.239 and 0.033 for *E. gracilis*, and 0.031 and −0.243 for *E. xingi*, respectively (Table 2), suggesting that the chain asymmetry was reversed [33].

### 3.2. Protein-Coding Genes, Composition, and Evolutionary Rates

**Figure 3:**
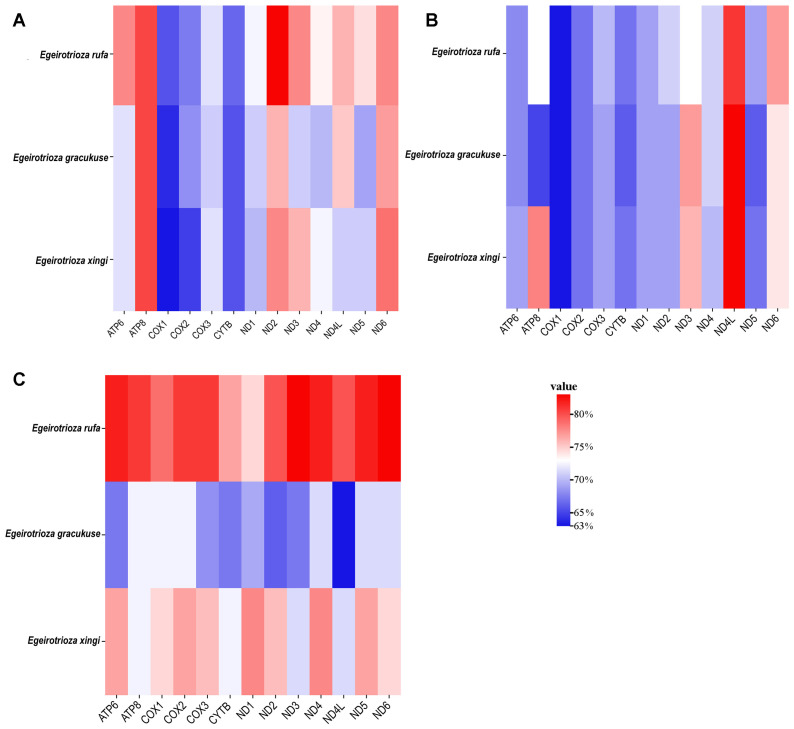
Difference in AT contents of protein-coding genes of *Egeirotrioza* mitochondrial genomes. (**A**) First codon positions; (**B**) second codon positions; (**C**) third codon positions.

There are no remarkable differences in the sizes of tRNAs, PCGs, and rRNAs among each species when compared. The PCG lengths of the three newly obtained species are 10,825 (*E. rufa*), 10,822 (*E. gracilis*), and 10,818 bp (*E. xingi*). All newly obtained mitochondrial genomes exhibit a negative GC-skew and AT-skew; the GC-skew values are −0.08 (*E. rufa*), −0.11 (*E. gracilis*), and −0.09 (*E. xingi*), while the AT-skew values are −0.13 (*E. rufa*), −0.14 (*E. gracilis*), and 0.13 (*E. xingi*) (Table 2). The AT contents (%) are 78.34 (*E. rufa*), 73.55 (*E. gracilis*), and 75.80 (*E. xingi*), and the GC contents (%) are 21.65 (*E. rufa*), 26.45 (*E. gracilis*), and 24.19 (*E. xingi*) (Table 2). Compared with published data, we found that the AT content of the third codon positions was higher than that of the first and second positions in PCGs (Figure 3). 

Similar to insect mitochondria, the three newly obtained mitochondrial genomes’ PCGs started with ATN [2,3,31]. However, diverse start codons were discovered: COI used ATG as the start codon in three species; the *ND3* gene used ATT in two species and ATA in one species; *ND4L* used TTG in three species; *ND5* used ATC in one species and ATT in two species; *ND2* used ATG in one species and ATA in two species, etc. The codon sizes of the three newly sequenced species were 1764 (*E. rufa*), 1303 (*E. gracilis*), and 1290 (*E. xingi*). Most PCGs in this group used TAA or TAG as the stop codon. However, *ND5*, *ND1*, and *COX2* in *Egeirotrioza* have an incomplete termination codon of TA or T; for example, that for *ND1* in *E. xingi* was TA and in *E. rufa* was T, and that for *COX2* in *E. xingi* and *E. rufa* was T. Incomplete termination codons of PCGs are frequently observed in insects and are typically completed through polyadenylation following the excision of the downstream tRNA gene [34,35,36].

**Figure 4:**
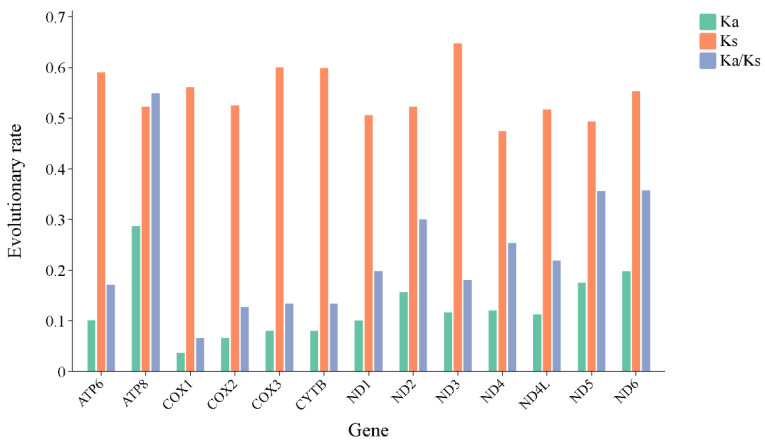
Evolution rates of 13 PCGs of *Egeirotrioza*. Ka refers to non-synonymous nucleotide substitutions, Ks refers to synonymous nucleotide substitutions, and Ka/Ks refers to the selection pressure of each PCG. The abscissa represents the 13 PCGs, and the ordinate represents the Ka/Ks values.

The Ka/Ks value (ω) is commonly employed to gauge the rate of sequence evolution driven by natural selection [37]. The Ka/Ks analysis results showed that all 13 PCGs have a value of less than one, ranging from 0.07 (*COX1*) to 0.55 (*ATP8*) (Figure 4), and the evolution rate is as follows: *ATP8* > *ND6* > *ND5* > *ND2* > *ND4* > *ND4L* > *ND1* > *ND3* > *APT6* > *CYTB* > *COX3* > *COX2* > *COX1*. This indicates that each gene has undergone purifying selection, and some genes such as *ATP8* and *ND6* experienced relatively relaxed selection pressure. The DNA barcoding gene *COX1* underwent the strongest purifying selection, which is consistent with previous studies of psyllids [6].

The 22 tRNAs ranged from 58 to 70 bp in length. The AT contents (%) of the newly obtained mitochondrial genomes are as follows: 79.45 (*E. rufa*), 78.17 (*E. gracilis*), and 79.60 (*E. xingi*). All the newly assembled mitochondrial genomes exhibit positive AT-skew and GC-skew; the AT-skew values are 0.013 (*E. rufa*), 0.024 (*E. gracilis*), and 0.035 (*E. xingi*), and the GC-skew values are 0.145 (*E. rufa*), 0.145 (*E. gracilis*), and 0.154 (*E. xingi*) (Table 2). The rRNA lengths are as follows: 1892 (*E. rufa*), 1891 (*E. gracilis*), and 1898 (*E. xingi*). The AT contents (%) are 79.08 (*E. rufa*), 76.95 (*E. gracilis*), and 78.60 (*E. xingi*). The AT-skew values of all mitochondrial genomes are negative (−0.035 to −0.016), while the GC-skew values are positive (0.0314 to 0.0357) (see Table 2).

### 3.3. Nucleotide Diversity and Codon Usage

**Figure 5:**
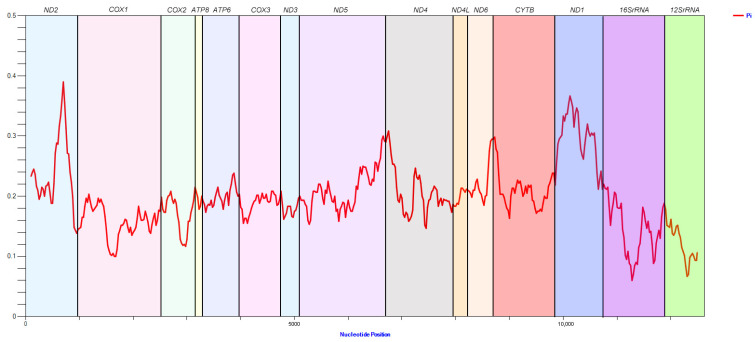
Sliding window analysis of 13 PCGs and 2 rRNAs in the mitochondrial genomes of 3 species of Triozidae. The red line represents the nucleotide diversity (Pi) value (window size = 200 bp, step size = 20 bp).

**Figure 6:**
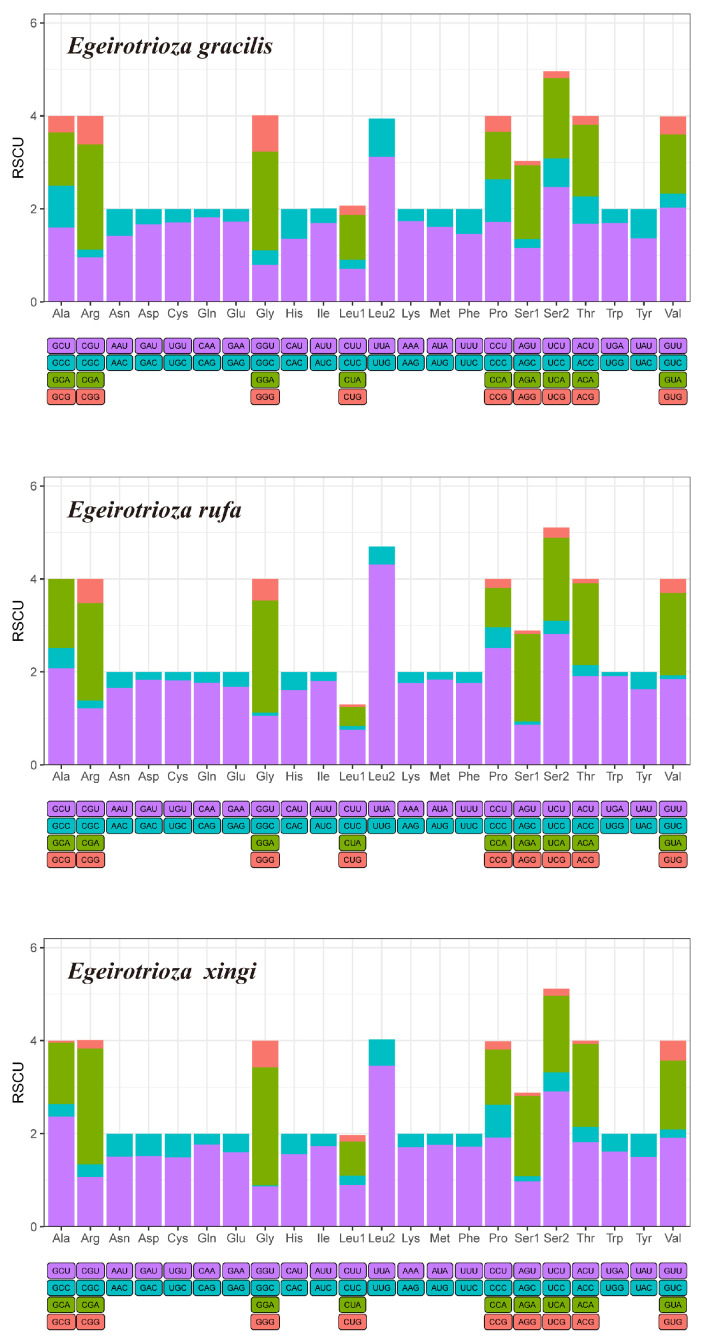
Relative synonymous codon usage (RSCU) of mitochondrial protein-coding genes of 3 Triozidae species. The *X*-axis shows different amino acids, and the *Y*-axis shows the RSCU value (the number of times a certain synonymous codon is used/the average number of times that all codons encoding the amino acid are used).

The sliding window analysis showed that the nucleotide diversity (Pi) of the 13 PCGs in *Egeirotrioza* is highly variable, with the highest Pi obtained for *ATP8* (0.390) followed by *ND1* (0.367) and *ND4* (0.280), and the lowest Pi for *COX1* (0.100) (Figure 5). The relative synonymous codon usage (RSCU) patterns exhibited by the three mitochondrial genomes are largely similar, as depicted in Figure 6, which includes the RSCU values for all possible synonymous codons corresponding to the 22 amino acids and 62 available codons used in the 13 PCGs of *Egeirotrioza* species. Among the *Egeirotrioza* species, UUA is the preferred codon, while Leu2, Ile, Phe, and Mte are the most frequently utilized amino acids. When considering the three species, *E. gracilis* and *E. xingi* favor the codons AUU, UUA, UUU, and AUA, whereas *E. rufa* prefers the codons UUA, AUU, UUU, and AUA. Prior to this study, the genus *Egeirotrioza* never had any molecular markers published in GenBank and BOLD systemv4. Our results on nucleotide diversity show that the *Egeirotrioza COX1* gene can be used as an effective molecular marker for classification. This study has generated barcode reference data for *Egeirotrioza* species in order to use DNA barcoding as a rapid tool for accurate identification of the psyllid to aid phytosanitary measures. Meanwhile, the published mitochondrial genome of *Egeirotrioza* also facilitates the application of multi-marker DNA meta-barcoding technology for rapid pest monitoring. Codons ending in A/T are used more frequently, and the AT content at the third codon position is higher than that at the first and second codons, indicating that the third codon is more vulnerable to AT alterations [38].

### 3.4. Phylogenetic Relationships

**Figure 7:**
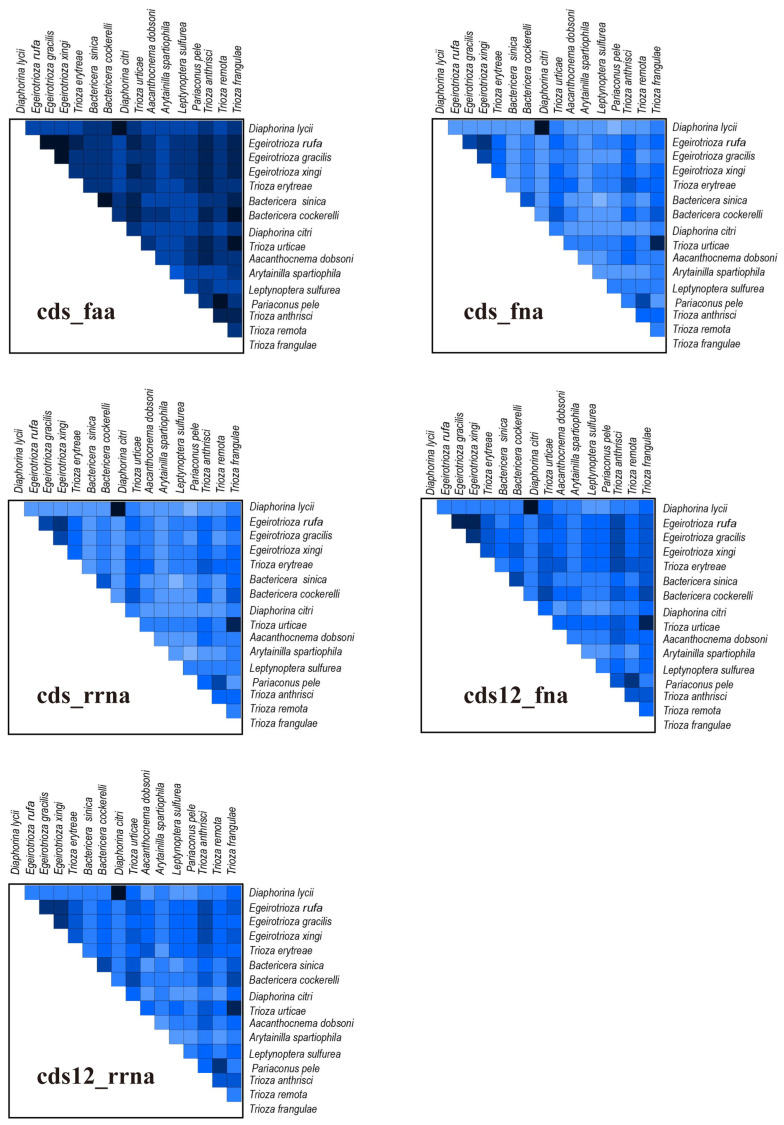
Heterogeneity analysis for different matrices. Colored squares represent pairwise Aliscore values. Score values range from −1 (indicating fully random similarity, dark blue) to +1 (indicating non-random similarity).

**Figure 8:**
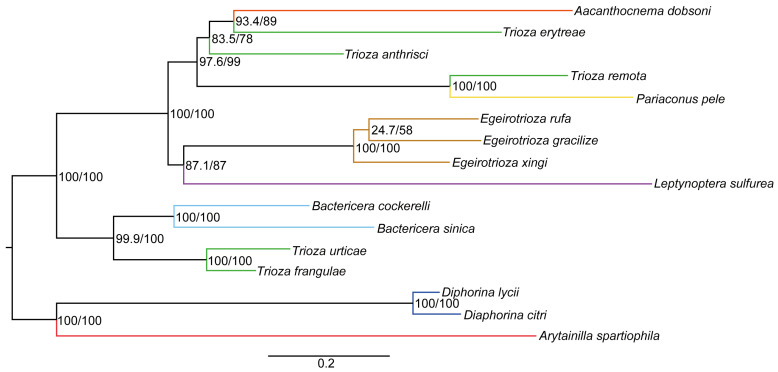
Phylogenetic trees of *Egeirotrioza* based on the cds_faa matrix with the PMSF model in IQ-TREE. Support values on nodes indicate r SH-aLRT/UFBoot2, respectively.

The heterogeneous divergence analysis using AliGROOVE found that the cds_faa matrix exhibited lower heterogeneity than the cds_fna, cds12_fna, cds12_rrna, and cds_rrna matrices (Figure 7). We used a supermatrix including cds_faa (5375 sites), cds_fna (7148 sites), cds_rrna (12,580 sites), cds12_fna (7148 sites), and cds12_rrna (9006 sites) to reconstruct the phylogenetic relationship of Triozidae based on BI and ML methods. The two approaches using these five matrices generated five BI and six ML trees, respectively (Figure 8 and Figures S1–S10).

The phylogenetic analyses using all the matrices produced highly consistent topologies, thus supporting the monophyly of *Egeirotrioza*. The phylogenetic relationships show that *Egeirotrioza* is closely related to *Leptynoptera* (Figure 8). The genus *Evegeirotrioza* was established by Li [10]. Subsequently, Burckhardt and Ouvrard [11] proposed the synonymy of *Evegeirotrioza* with *Egeirotrioza* based on the morphological character of the genital plate, which is conical when viewed from the side. *Evegeirotrioza* was morphologically considered to be synonymous with *Egeirotrioza*, but there was no evidence of molecular correlation. In our phylogenetic studies, we formed the structure (*Egeirotrioza xingi* + (*Evegeirotrioza rufa* + *Evegeirotrioza gracilis*)) (Figure 8 and Figures S1–S10). *Evegeirotrioza* has been well restored to the taxa of *Egeirotrioza*, so we reject *Evegeirotrioza* at both morphological and molecular levels and restore the monophyly of *Egeirotrioza* [39]. As *Egeirotrioza* has never been reported as a molecular marker, the genus was not included in previous phylogenetic studies based on molecular morphology.

Trioza is considered by most authors as an artificial receptacle for species not showing any particular morphological modifications. In our results, the position of *Trioza* (five species) was located in four groups, and the relationships of *Leptynoptera* and *Bactericera* are highly consistent with previous studies [6]. In Triozidae, a species-rich, probably monophyletic family, most of the genera are ill-defined and artificial, and the phylogenetic relationships between genera remain largely unknown. Here, we have provided the mitochondrial DNA of *Egeirotrioza*, the first molecular analysis of its phylogenetic position, and also new fundamental data on the phylogenetic relationships of Psyllidae.

Due to the limited samples, the position of the genus *Trioza* was unclear and it also did not support the monophyly of *Trioza*, which was located in four groups (Figure 8). Additional taxon sampling, data, and analyses are necessary to resolve this ambiguity in future studies. For instance, low-coverage sequences are often utilized to extract more molecular markers to reconstruct stable phylogenetic relationships, as successfully demonstrated in other groups [40,41,42].

## 4. Conclusions

Three mitochondrial genomes of *Egeirotrioza* were obtained, including two complete mitochondrial genomes and one linear mitochondrial genome. The newly sequenced mitochondrial genomes exhibit similar structural features and nucleotide compositions to the previously published data of Triozidae. The nucleotide diversity (Pi) analysis showed that the diversity of *ATP8* is the highest, and that of *COX1* is the lowest. The three mitochondrial genomes display similar relative synonymous codon usage (RSCU) patterns. In adding published data, we could also reconstruct the phylogenetic relationships among Triozidae. In our results, the monophyly of *Egeirotrioza* was well supported, while the position of the genus *Trioza* was unclear due to the limited samples.

## Figures and Tables

**Table 1:** Collected information of newly sequenced species in this study.

Species	Location	Longitude and Latitude	Elevation (m)	Data	Collector
*Egeirotrioza gracilis*	Luntai County, Xinjiang, China	84.3081° E, 41.5911° N	931.4	27.V.2023	Chen-Hong Wang
*Egeirotrioza rufa*	Luntai County, Xinjiang, China	84.2050° E, 41.2537° N	928.1	26.V.2023	Jin-Ling Wang
*Egeirotrioza xingi*	Luntai County, Xinjiang, China	84.2050° E, 41.2537° N	931.4	27.V.2023	Chen-Hong Wang

**Table 2:** Nucleotide compositions of three newly sequenced species in this study.

Species	Whole Genome					PCG					tRNA					rRNA					CR				
	Length	AT%	AT-	GC%	GC-	Length	AT%	AT-	GC%	GC-	Length	AT%	AT-	GC%	GC-	Length	AT%	AT-	GC%	GC-	Length	AT%	AT-	GC%	GC-
	(bp)		Skew		Skew	(bp)		Skew		Skew	(bp)		Skew		Skew	(bp)		Skew		Skew	(bp)		Skew		Skew
*Egeirotrioza rufa*	15,830	76.920	0.010	23.06	−0.154	10,825	78.343	−0.134	21.64513	−0.080	1377	79.450	0.013	20.55	0.145	1892	79.080	−0.019	20.92	0.314	1764	71.090	−0.065	28.74	0.014
*Egeirotrioza gracilis*	15,355	74.460	0.033	25.53	−0.23854	10,822	73.553	−0.135	26.44744	−0.110	1361	78.170	0.024	21.82	0.145	1891	76.975	−0.016	23.03	0.331	1303	82.880	0.006	16.96	−0.177
*Egeirotrioza xingi*	15,301	76.4	0.031	23.63	−0.243	10,818	75.8	−0.131	24.19436	−0.094	1367	79.6	0.035	20.41	0.154	1898	78.6	−0.035	21.4	0.357	1249	84.7	−0.009	15.14	−0.100

## Data Availability

Regarding the availability of DNA sequences, the new mitochondrial genomes are deposited in GenBank (NCBI), and the accession numbers are PP471964-PP471966.

## Supplementary Materials

The following supporting information can be downloaded at: https://www.mdpi.com/article/10.3390/genes15070842/s1, Figure S1: Bayesian inference phylogenetic tree of *Egeirotrioza* based on the analysis of cds_faa with a GTR + CAT model in PhyloBayes. Support values on nodes indicate Bayesian posterior probabilities; Figure S2: Maximum likelihood phylogenetic tree of Egeirotrioza based on the analysis of cds_faa with the Partition model in IQ-TREE. Support values on nodes indicate SH-aLRT/UFBoot2, respectively; Figure S3: Bayesian infer-ence phylogenetic tree of Egeirotrioza based on the analysis of cds_fna with a GTR + CAT model in PhyloBayes. Support values on nodes indicate Bayesian posterior probabilities; Figure S4: Maximum likelihood phylogenetic tree of Egeirotrioza based on the analysis of cds_fna with the Partition model in IQ-TREE. Support values on nodes indicate SH-aLRT/UFBoot2, respectively; Figure S5: Bayesian inference phylogenetic tree of Egeirotrioza based on the analysis of cds_rrna with a GTR + CAT model in PhyloBayes. Support values on nodes indicate Bayesian posterior probabilities; Figure S6: Maxi-mum likelihood phylogenetic tree of Egeirotrioza based on the analysis of cds_rrna with the Partition model in IQ-TREE. Support values on nodes indicate SH-aLRT/UFBoot2, respectively; Figure S7: Bayesian inference phylogenetic tree of Egeirotrioza based on the analysis of cds12_fna with a GTR + CAT model in PhyloBayes. Support values on nodes indicate Bayesian posterior probabilities; Figure S8: Maximum likelihood phylogenetic tree of Egeirotrioza based on the analysis of cds12_fna with the Partition model in IQ-TREE. Support values on nodes indicate SH-aLRT/UFBoot2, respectively; Figure S9: Bayesian inference phylogenetic tree of Egeirotrioza based on the analysis of cds12_rrna with a GTR + CAT model in PhyloBayes. Support values on nodes indicate Bayesian posterior probabilities; Figure S10: Maximum likelihood phylogenetic tree of Egeirotrioza based on the analysis of cds12_rrna with the Partition model in IQ-TREE. Support values on nodes indicate SH-aLRT/UFBoot2, respectively.
