# Supplementary material for: The First Three Mitochondrial Genomes for the Characterization of the Genus Egeirotrioza (Hemiptera: Triozidae) and Phylogenetic Implications
Source: Genes (Basel). 2024 Jun 26;15(7):842. doi: 10.3390/genes15070842 (PMC11275608; doi:10.3390/genes15070842)
Supplement: Supplementary file 1 [file genes-15-00842-s001.zip › genes-3029017-supplementary.pdf]

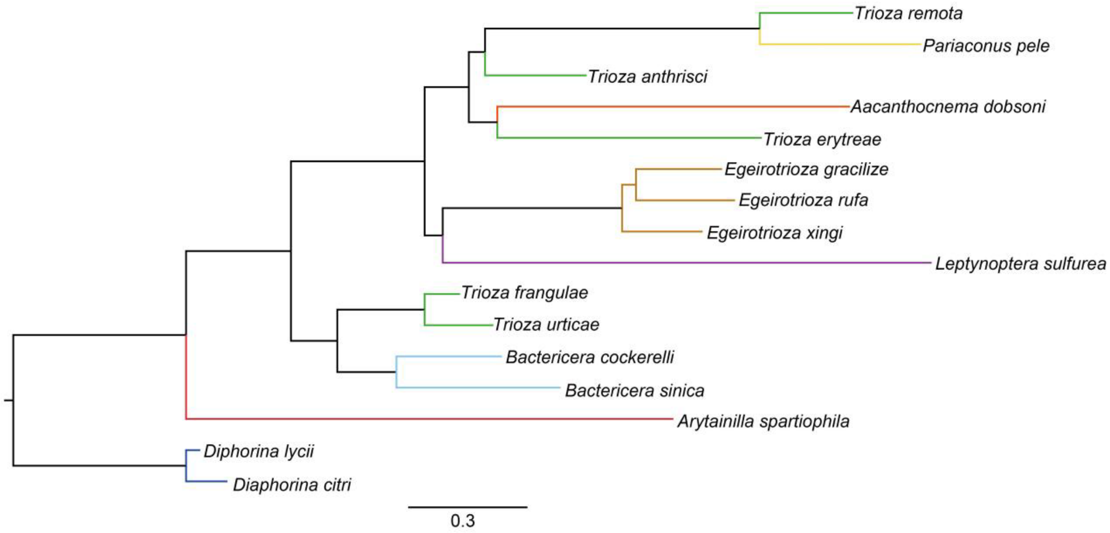

Figure S1. cds_faa_BI.

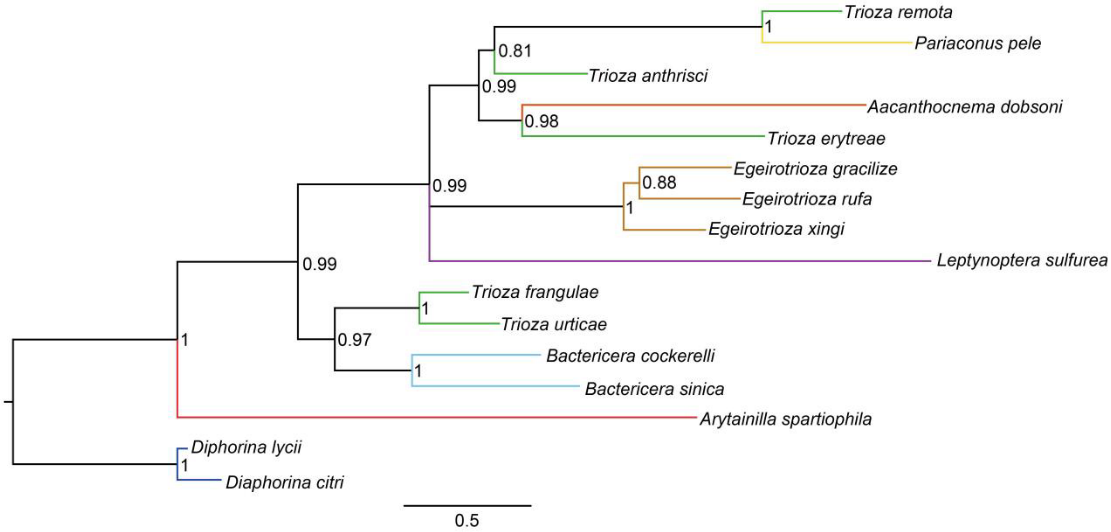

Figure S2. cds_fna_BI.

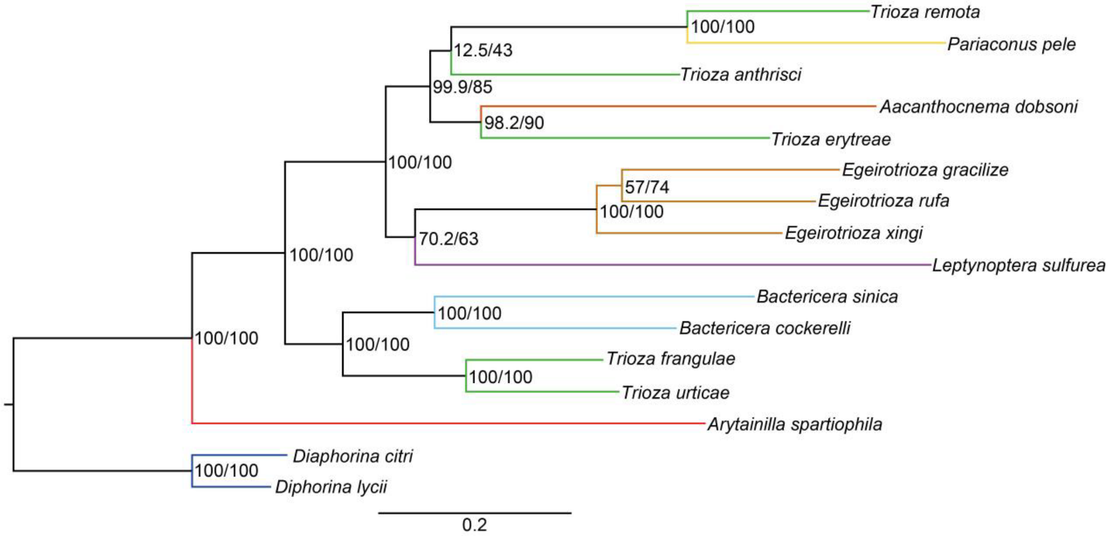

Figure S3. cds_fna_partition.

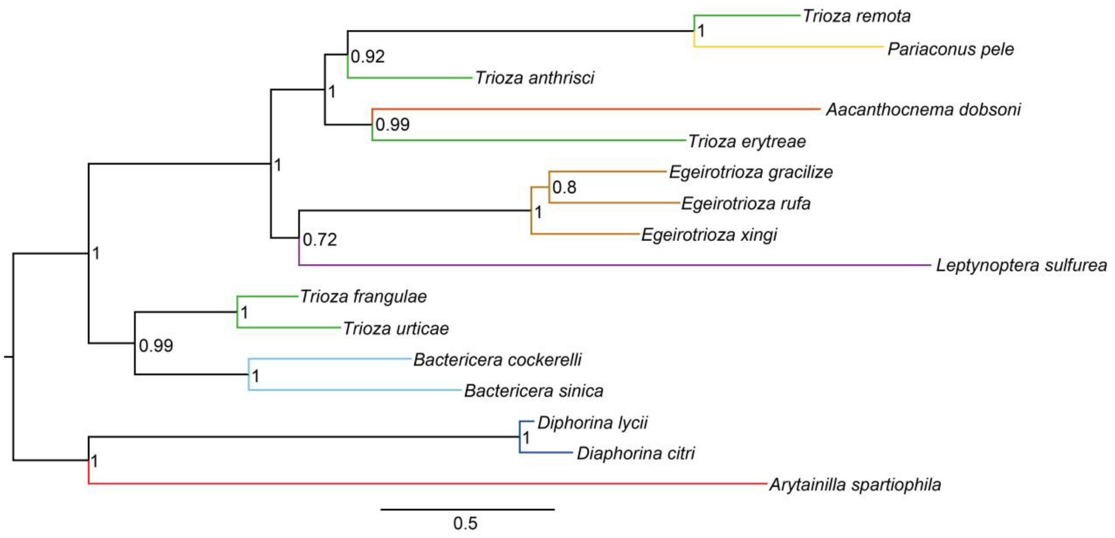

Figure S4. cds_rrna_BI.

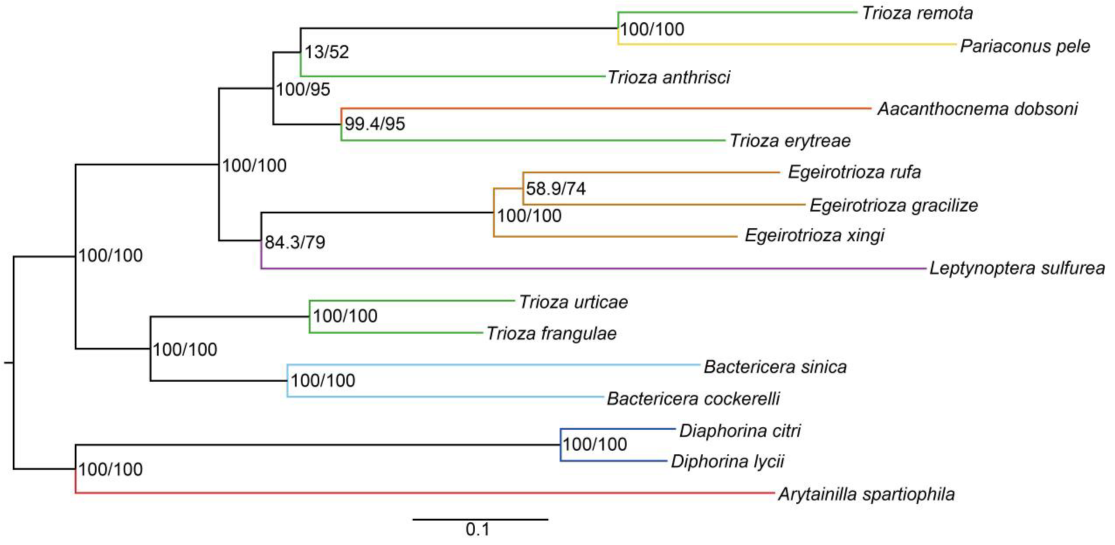

Figure S5. cds_rrna_partition.

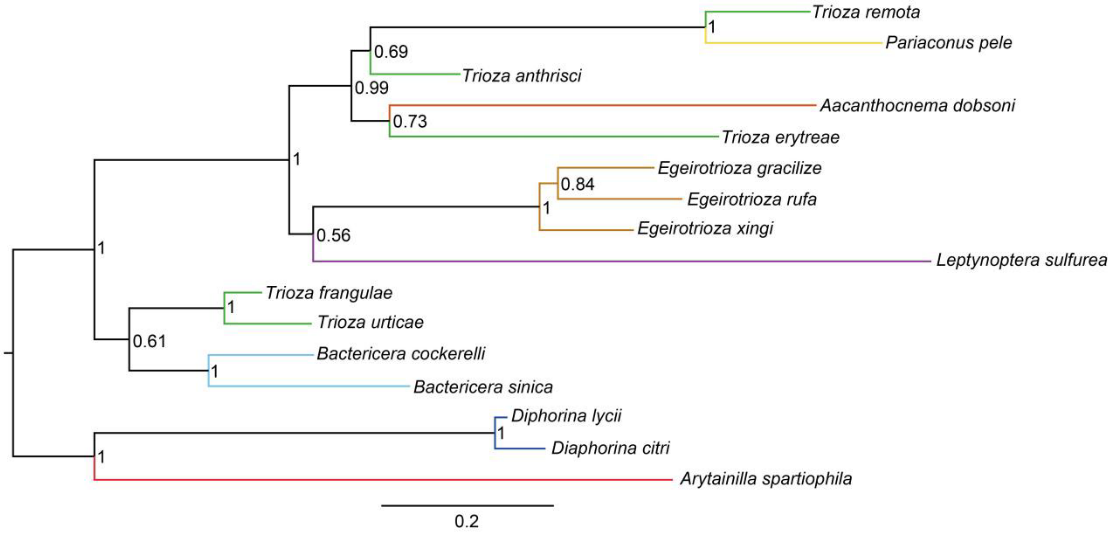

Figure S6. cds12_fna_BI.

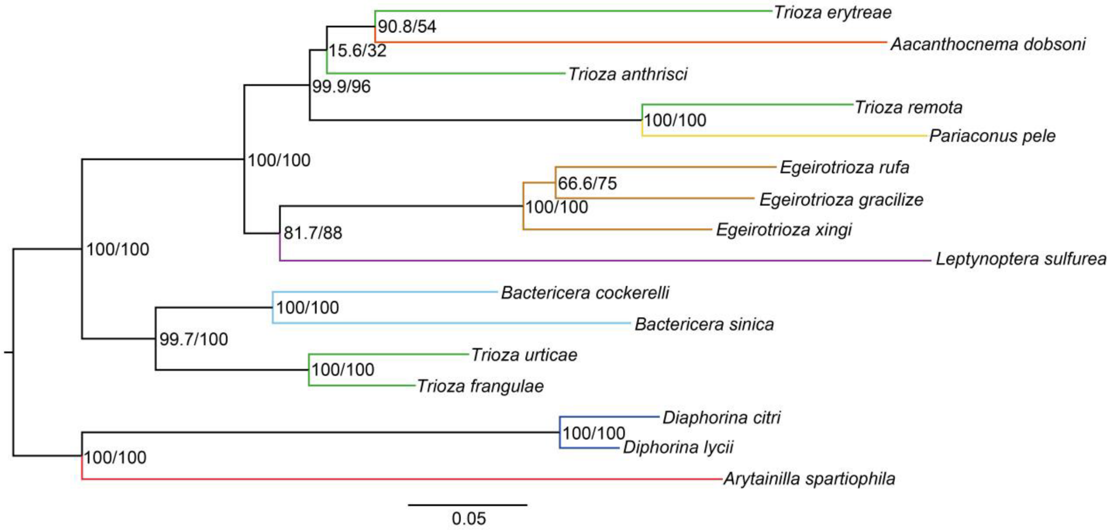

Figure S7. cds12_fna_partition.

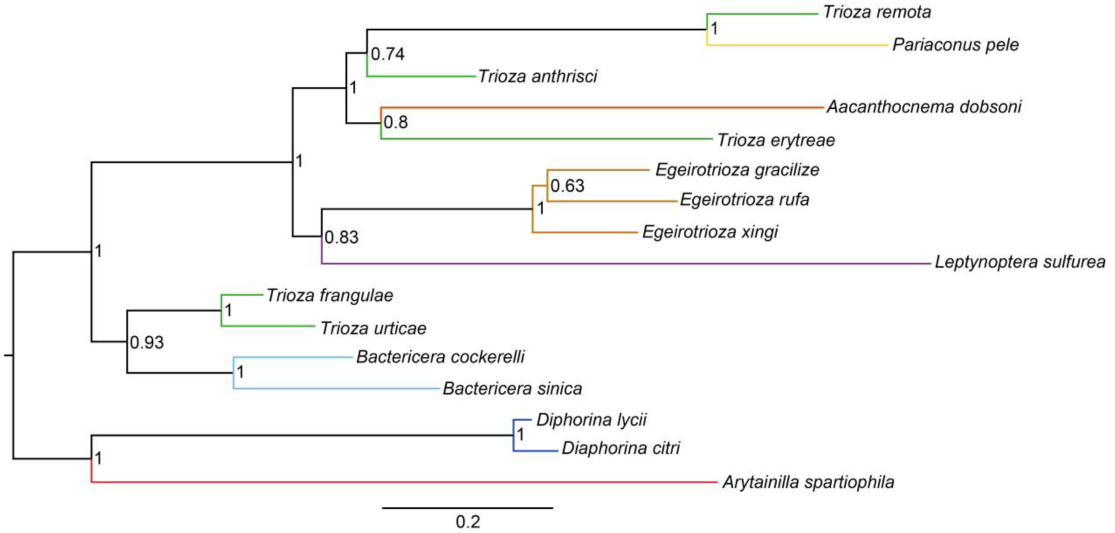

Figure S8. cds 12_rrna_BI.

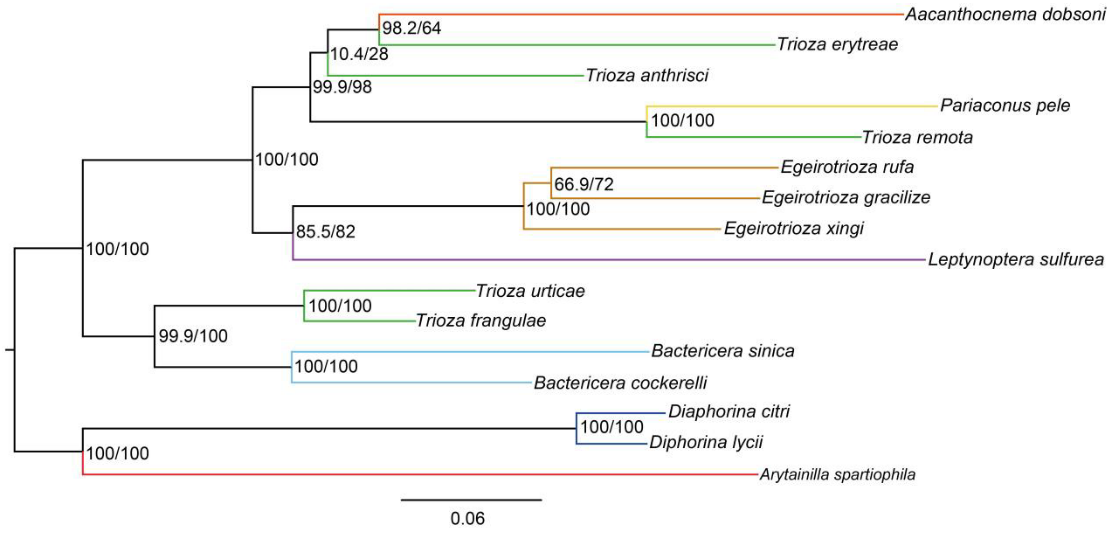

Figure S9. cds12_rrna_partition.
